# Impaired Executive Functioning of Sexual Assault Survivors with Acute Stress Disorder

**DOI:** 10.3390/jcm7100362

**Published:** 2018-10-16

**Authors:** Su Mi Park, Jung-Seok Choi, Ji Sun Lee, Jun-Young Lee, Saerom Lee, Hee Yeon Jung

**Affiliations:** 1 Department of Psychiatry, SMG-SNU Boramae Medical Center, Seoul 07061, Korea; lovessum12@snu.ac.kr (S.M.P.); choijs73@gmail.com (J.-S.C.); jisune87@gmail.com (J.S.L.); benji@snu.ac.kr (J.-Y.L.); quf1316@gmail.com (S.L.); 2 Department of Clinical Medical Sciences, Seoul National University College of Medicine, Seoul 03080, Korea; 3 Department of Psychiatry and Behavioral Science and Institute of Human Behavioral Medicine, National University College of Medicine, Seoul 03080, Korea; 4 Department of Psychiatry and Neuroscience Research Institute, Seoul National University College of Medicine, Seoul 03080, Korea

**Keywords:** acute stress disorder, sexual assault, executive functioning

## Abstract

This study aimed to examine the executive functioning of survivors exposed to recent sexual assaults. Twenty-seven female rape survivors who met the criterion for acute stress disorder (ASD) were enrolled and completed the assessment within 4 weeks after the traumatic experience. Additionally, 25 age-matched female health controls (HC) never exposed to such a traumatic event were enrolled. The assessments evaluated psychiatric symptoms including post-traumatic stress symptoms, depression, anxiety, and trait and state anger; general intelligence indexed by intellectual quotient (IQ); and executive functioning including set-shift/attention, planning, spatial working memory, and inhibition using the Cambridge Neuropsychological Test Automated Battery. The ASD group showed higher levels of depression, anxiety, and state anger, and lower IQ than the HC group. The ASD group also showed global impairment of executive functioning for set-shifting, attention, planning, and response inhibition compared to the HC group. Rather than being associated with low IQ and education levels, these results could be from trauma-related effects in survivors with ASD. Additionally, the state anger level was related to survivors’ deficient executive functioning. The findings indicate the importance of providing assessment and intervention efforts to sexual assault survivors soon after the trauma occurs.

## 1. Introduction

A traumatic stress experience has short- and/or long-term physical and psychological impacts. In the current version of the Diagnostic and Statistical Manual of Mental Disorders (DSM-5) [1], stress and trauma-related disorders include two types of psychiatric disorders that develop after experiencing an extremely traumatic event (e.g., direct or indirect exposure to life-threatening events and sexual assaults): post-traumatic stress disorder (PTSD) and acute stress disorder (ASD). PTSD is diagnosed one month after the traumatic event, and all four clusters of criteria—intrusive/re-experience, avoidance, negative change in cognition and mood, and alteration in arousal and reactivity—are needed for its diagnosis. ASD is diagnosed within one month after experiencing the trauma. ASD has five clusters of symptoms: intrusion, negative mood, dissociation, avoidance, and arousal. Many ASD symptoms are similar to those of PTSD; however, ASD and PTSD differ in terms of the clusters of symptoms required for diagnosis. ASD diagnosis requires meeting the criteria for at least 9 of the 14 symptoms and this method of diagnosis is introduced in the DSM-5. As diagnosing ASD involves identifying it within a certain time limit, which can be challenging for researchers, few studies have examined the incidence of ASD as compared to PTSD.

Sexual assault is a type of prevalent traumatic stress experience that affects the mental health of survivors. One large-scale epidemiological study found that rape and molestation were identified as the most upsetting traumatic event (49%) in women with PTSD when compared with other traumatic events, including accidents, being threatened with a weapon, and physical abuse [2]. The lifetime prevalence of PTSD for women who have been sexually assaulted is 50%, which is much higher than the general prevalence of PTSD, which is 6.8% [3,4,5]. Moreover, one study that examined PTSD symptoms among women who were raped found that 94% experienced these symptoms during the two weeks immediately following the rape [6]. According to the theory of rape trauma syndrome (RTS), a rape survivor experiences psychological trauma that includes disruptions to normal physical, emotional, cognitive, and interpersonal behaviors, overlapping with disruptions associated with ASD and PTSD [7,8]. RTS identifies three stages of psychological trauma that rape survivors undergo: the acute stage, the outer adjustment stage, and the renormalization stage. The outward adjustment stage may last from several months to many years, even a person’s whole lifetime, after the rape. It has also been found that rape survivors are at high risk for developing substance use disorders, major depression, anxiety disorder, and eating disorders [9,10]. Many controversies on whether ASD predicts PTSD exist [11], but regardless, it is important to detect rape-related symptoms soon after the event and conduct appropriate therapeutic intervention to prevent the chronicization of PTSD and other mental disorders.

Cognitive function interacts with emotional change and has been identified as a factor playing an important role in developing, maintaining, and treating PTSD and other psychiatric disorders [12]. Numerous previous studies have focused on the abnormal memory function and fear conditioning of PTSD; these phenomena are characterized by reduced hippocampal and amygdala activity [13,14,15,16]. More recently, the role of executive function in PTSD has been receiving attention from researchers [17]. Executive function plays a key role in high-order cognitive function and contains attention/working memory—the active maintenance and manipulation of information in one’s mind over a short period of time—sustained attention/inhibitory function, flexibility/switching, and planning. Some studies have found deficits in these aspects of executive functioning in participants with PTSD [18,19,20]. Such abnormality in executive functioning could be a predisposition to PTSD and/or consequence of it [18,21]. For instance, difficulties in attentional disengagement from traumatic stimuli and inhibition could be associated with hyper-arousal and intrusive memory symptoms as well as diminished concentration. Neuroimaging studies have reported consequences from PTSD that include the disrupted functional change in frontal regions such as the anterior cingulate cortex (ACC) and prefrontal cortex (PFC), including the inferior PFC (IFC), ventrolateral PFC, and dorsolateral PFC [22,23].

Cognitive function soon after trauma occurs seems to be crucial for the psychological health of survivors. A cognitive deficit may interfere with psychological trauma recovery, a process that requires re-learning and adaptation. However, few studies on neurocognitive function in those with ASD have been conducted compared to PTSD. Brandes et al. [24] found that survivors from various recent traumatic events with high initial levels of PTSD symptoms had impaired attention and immediate recall of visual information and a lower intellectual quotient (IQ) than those with low initial symptoms. LaGarde et al. [25] found a disturbed fronto-temporal system function in trauma-exposed individuals with acute PTSD. Moreover, the study of rape survivors is even more challenging due to the high rate of survivors reluctant to disclose their victimization and seek help because of shame and fear of social stigma. One study on survivors of recent sexual assault with a multimodal approach found that, compared to healthy controls (HCs), survivors showed a deficit in cognitive flexibility and that their dysfunctions in the dorsal ACC and the cerebellum may change when presented with emotional stimuli [26]. However, this study did not include the assessment of IQ levels, which have been shown to be related to a person’s reaction to stress and might have confounding effects on executive functioning [27]. This previous study [26] also did not comprehensively explore the relationship between executive function and acute stress sub-symptoms or psychiatric symptoms, though the survivors exhibited increased depressive and anxiety symptoms, which might be related to cognitive deficits [28,29].

The present study focused on the executive functioning of survivors exposed to recent sexual assault, its relationship to stress-related symptoms, and its potential effect on survivors’ prognoses. The first hypothesis of this study was that sexual assault survivors would exhibit disrupted executive functioning, including attention/working memory, set-shift/flexibility, inhibitory functioning, and planning, compared to a control group never exposed to such a traumatic event. To avoid the possible confounding effects of global intelligence on executive function, IQ was also assessed. The second hypothesis was that the survivors’ executive dysfunction (attention/working memory, set-shift/flexibility, inhibitory functioning, and planning) seen immediately after the rape would be associated with stress-related sub-symptoms and psychiatric conditions (depression, anxiety, and anger).

## 2. Method

### 2.1. Participants

The institutional review board (IRB) of the SMG-SNU Boramae Medical Center approved the study protocol (IRB number of the original study: 06-2012-114; IRB number of the subsequent study: 16-2017-66). All subjects provided written informed consent.

After collaboration with the emergency center and the sexual assault referral center (Seoul South Sunflower Center) in the SMG-SNU Boramae Medical Center, 27 female sexual assault survivors who visited the department of psychiatry within 4 weeks after the event were included in this study. Diagnosis of ASD was made according to the DSM-5 guidelines, and the participants’ present psychiatric comorbidity and history of psychiatric disorder were checked using the Mini International Neuropsychiatric Interview-Plus (MINI-Plus) [30,31]. Psychiatric and other medical illness interviews were administered by psychiatrists and psychologists. All survivors met the criteria for ASD, and of those, 24 were exposed to complete rape and three to intended rape. Two survivors had a history of psychiatric disorder (depression *n* = 2). Additionally, 25 age-matched HCs with no experience of sexual assault or any kind of extreme traumatic event were included. To screen for the history of psychiatric disorder, MINI-Plus was also used. Regardless of the group, participants with a history of head injury or neurological disease (seizures, tumor, etc.), psychotic disorder, mental retardation, or substance use disorder were excluded.

### 2.2. Assessments

#### 2.2.1. PTSD Symptom Scale-Self Report (PSS-SR)

Self-reported severity of acute stress-related syndromes of the ASD group was measured with the PSS-SR [32]. Though the PSS-SR was originally developed to assess the severity of PTSD symptoms within the past week, it has been used to measure trauma exposure-related syndromes such as acute stress disorder [33]. PTSD symptom severity is rated for 17 items with a 4-point Likert scale from 0 to 3; scores per symptom add up to a total severity score ranging from 0 to 51, with higher scores suggesting higher PTSD severity. Additionally, subtypes of symptoms were also categorized as the following: re-experience (5 items), avoidance (2 items), numbing (5 items), hyper-arousal (5 items), and dysphoria (8 items) [34,35]. The internal consistency (Cronbach’s alpha) in this study was 0.89. The PSS-SR was conducted only for the ASD group and not for the HC group.

#### 2.2.2. Korean Beck Depression Inventory-II (BDI-II)

This is a 21-item self-report questionnaire with a 4-point Likert scale (0–3) used to assess the degree of depression experienced during the past week [36,37]. Cronbach’s alpha of the BDI-II in this study was 0.95.

#### 2.2.3. Korean Beck Anxiety Inventory (BAI)

This is a 21-item self-report questionnaire with a four-point Likert scale (0–3); it was also used to assess the degree of anxiety experienced during the past week [38,39]. Cronbach’s alpha of the BAI in this study was 0.98.

#### 2.2.4. Korean State Trait Anger Expression Inventory (STAXI)

STAXI is a self-reported questionnaire with a four-point Likert scale (1–4), translated by Chon, et al. [40] to measure anger levels and anger expression [41]. In this study, the state anger (STAXI-S) and trait anger (STAXI-T) subscales including 10 items for each were selected. Cronbach’s alpha of STAXI in this study was 0.90.

#### 2.2.5. Korean Wechsler Adult Intelligence Scale–Fourth Edition (K-WAIS–IV)

Cognitive ability and IQ were assessed using the K-WAIS-IV [42,43]. As individuals with ASD who enrolled first five were assessed with an earlier version of K-WAIS [44], we conducted the Mann–Whitney U test on the IQ score of the ASD group to test for any differences in the versions, which we did not find (*p* > 0.05).

#### 2.2.6. Cambridge Neuropsychological Test Automated Battery (CANTAB)

Neuropsychological functioning was measured using the CANTAB [45] (http://www.camcog.com, Cambridge Cognition Ltd., Cambridge, UK). This series of computerized tasks was run on an Acorn BBC Master 128 microcomputer with a high-resolution Microvitec (Acorn Computers Ltd., Cambridge, UK) 12-inch VDU and a Microvitec Touchtec 501 touch-sensitive screen. Participants sat at a comfortable height approximately 0.5 m from the monitor. They were instructed to respond to stimuli by touching the screen or response pad. All participants performed the test in the same order. The test battery in this study consisted of the following tests (in order).

- Intra-Extra Dimensional Set Shift (IED): This test of rule acquisition and reversal, which is a computerized version of the Wisconsin Card Sorting Test, was used to assess cognitive flexibility/set-shift. Participants were required to pay attention to a reinforced stimulus (shapes: intra-dimensional shift) and subsequently shift to a previously irrelevant stimulus (lines: extra-dimensional shift). The test assessed participants’ ability to learn in a total of nine stages.
- Stockings of Cambridge (SOC): A variant of the classic Tower of London task, SOC was used to assess spatial planning. Participants were asked to mimic a display pattern with as few moves as possible. In the trial with the highest difficulty, participants have to move five times to solve the problem.
- Spatial Span (SSP): A visuospatial version of the digit span test, SSP was used to assess attention/working memory. Participants were instructed to mimic sequential flashing boxes in the order presented.
- Stop Signal Task (SST): This stop-signal response-inhibition test was used to assess the ability to inhibit a prepotent response. Participants were instructed to hit a press-pad as quickly and accurately as possible when an image of an arrow was shown, but to avoid hitting the pad when a beep was heard with the arrow.

### 2.3. Procedure

After informed consent was acquired, a screening interview was conducted. When all inclusion criteria were met, clinical measurements and neuropsychological tests were administered within a few days after enrollment. All the survivors with ASD completed the protocol in this study within 4 weeks after their exposure to sexual assault.

### 2.4. Statistical Analysis

Statistical analysis was conducted using R 3.4.4 (https://www.r-project.org). Group differences in demographic, clinical, and neuropsychological functioning were tested with a linear model or generalized linear model (GLM). Poisson distribution was adopted to the GLM analysis on the variables with number of errors (e.g., CANTAB IED total error), and the other variables that did not meet the normal distribution were analyzed with gamma or quasi family. GLM with adjustment for education and for IQ was additionally conducted in order to avoid the possible confounding effects on executive functioning. The significance level with a two-tailed *p*-value to measure statistical differences between groups was set at <0.05.

Spearman’s correlation analysis among variables was conducted for data including both groups. False discovery rate (FDR) based on a Benjamini and Hochberg method was used for multiple corrections [46].

## 3. Results

### 3.1. Group Differences in the ASD and HC Groups

#### 3.1.1. Demographic and Clinical Characteristics

Table 1 presents the descriptive statistics for demographic and clinical characteristics of the ASD and HC groups. The ASD group showed shorter duration of education and lower level of IQ than did the HC group: *X*^2^ = 6.14, *p* = 0.0132 and *F* = 6.58, *p* = 0.0138, respectively. As the acute stressed state might affect the total IQ, we also applied the vocabulary subtest of K-WAIS-IV, which is known to be relatively less affected by variation in participants’ condition. Results revealed that the difference for the vocabulary score between the groups was not significant.

For the psychiatric symptom variables, the ASD group reported a significantly higher level of depression on the BDI-II, anxiety on the BAI, and state anger on the STAXI-S than the HC group: *X*^2^ = 63.28, *p* < 0.0001, *X*^2^ = 67.76, *p* < 0.0001, and *X*^2^ = 54.72, *p* < 0.0001, respectively. A significant difference between the groups was not found for trait anger measured using STAXI-T.

#### 3.1.2. Executive Functioning

**Figure 1:**
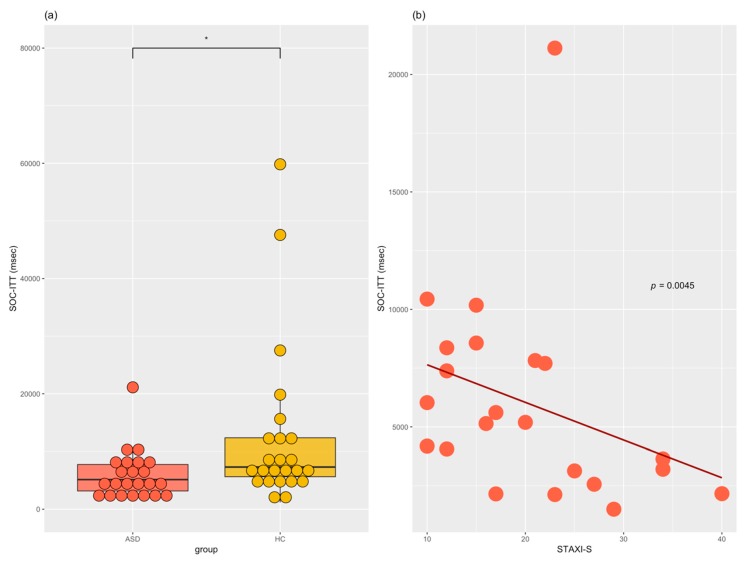
Executive functioning of ASD and HC. (**a**) Group differences for SOC-ITT; (**b**) Spearman’s correlation for STAXI-S and SOC-ITT in the ASD group; * *p* < 0.05; ASD = acute stress disorder; HC = healthy control; STAXI-S = State-Trait Anger Expression Inventory-state; SOC-ITT = Stockings of Cambridge-mean initial thinking time on five moves (msec).

Table 2 presents the descriptive statistics for executive performance of the ASD and HC groups. Compared to the HC group, the ASD group showed greater total numbers of errors in the IED measuring set-shifting and in the SSP measuring spatial attention/working memory: *X*^2^ = 13.72, *p* = 0.0002, *X*^2^ = 7.32, *p* = 0.0068, respectively. In the SOC test measuring planning, the ASD group showed a lower level of performance in problem-solving using a minimum number of moves and shorter latency for mean initial thinking time on trials using five moves compared to the HC group: *X*^2^ = 5.95, *p* = 0.0148, *X*^2^ = 7.42, *p* = 0.0064 (Figure 1a), respectively. In addition, the ASD group showed shorter reaction time for the SST measuring inhibition and for mean correct or incorrect reactions on go trials than the HC group: *X*^2^ = 7.76, *p* = 0.0053. The ASD group also showed a lower proportion of successful stops in the SST in the last half trials: *X*^2^ = 5.34, *p* = 0.0209. The group differences were not significant for IED total trials and SSP span length.

According to the results for group difference in education and global intellectual functioning, the effects of education and IQ on executive functioning were adjusted for in the further analysis. Results revealed that the group differences in the number of errors in IED and SSP still appeared significant after adjusting for either education and IQ (for errors in IED: adjusting for education *p* = 0.0182 and for IQ *p* = 0.0228; for errors in SSP adjusting for education *p* = 0.0013 and for IQ *p* = 0.0066). The lower reaction time during the SST of the ASD group than that of the HC group was also significant (adjusting for education *p* = 0.0191 and for IQ *p* = 0.0066). The difference between the groups regarding initial thinking time on five moves in the SOC was significant after adjusting for education; however, the group difference was found to be marginal but not significant after adjusting for IQ (adjusting for education *p* = 0.0199 and for IQ *p* = 0.0508). Differences in the groups were not found for SOC problem-solving using the minimum amount of moves nor for the proportion of successful stops in the last half trials in the SST after controlling for the effect of education or IQ (*p* > 0.05 for all).

### 3.2. Relationship among Factors

In all groups, the result of Spearman’s correlation analysis showed that the proportion of successful stops in the last half trials in SST was negatively correlated with the BAI score (*rho* = −0.39, *p* = 0.0071). IQ was positively correlated with the number of problems solved in a minimum amount of moves in the SOC (*rho* = 0.40, *p* = 0.0076).

In the ASD group, the mean initial thinking time in five moves in the SSP had significant negative correlation with the STAXI-S score (*rho* = −0.57, *p* = 0.0045; Figure 1b). Trends were found in the correlations corresponding to the reaction time of the SST and the score for the BDI-II; the number of problems solved in the SOC and the score for the BAI; and the number of problems solved in the SOC and IQ. However, these relationships were not significant after multiple corrections through FDR. There were no significant differences between the total PSS score and its subscales and variables of executive functioning assessed with CANTAB. Correlations for details including inter-domain variables of the ASD group are presented in Table S1 in the Supplementary Materials section.

## 4. Discussion

This study aimed to clarify the deterioration of executive functioning among female sexual assault survivors with ASD during the early stage after trauma. As expected, the main findings of our study indicate that sexual assault survivors with ASD who recently experienced trauma show disruptive executive functioning compared to the HC group with no trauma experience. The deficit in executive functioning of the ASD group appeared in most domains including attention/set-shifting, working memory, planning, and response inhibition. Compared to the HC group, the ASD group made more errors during tasks measuring attention/set shifting and working memory. Moreover, they displayed more incorrect or ineffective responses and insufficient response time in tasks measuring inhibition and planning. The poorer executive functioning of the ASD group was still found even after controlling for education or IQ.

The results of this study are comparable to those of a study reporting disrupted neurocognitive functioning in the later stage of trauma for survivors with PTSD [17]. Sexual assault survivors’ executive dysfunction in the acute stage after trauma in this study can be explained using various mechanisms. One is the immediate effect of extreme stress on the autonomic/endocrine reaction and brain function [47]. In chronic PTSD studies, it is difficult to determine whether changes in the brain are due to long-term effects of stress or changes due to other influences [48]. The present study can infer the possibility that acute stress may have affected the brain. Animal studies have found that acute stress impairs cognitive function in the PFC through a hyper dopaminergic mechanism [49,50]. Correspondingly, one human study demonstrated that experimentally induced acute stress in healthy women results in a reduction in the dorsolateral PFC activity related to the working memory, which may be explained by supraoptimal levels of catecholamines potentially in conjunction with elevated levels of cortisol and maladaptive hypothalamic-pituitary-adrenal (HPA) axis response [51].

The second reason for deficits in the ASD group may be related to an individual’s cognitive functioning predisposition before exposure to trauma. A recent study with HCs investigated how individual brain connectivity patterns affect an individual’s cognitive response while under acute stress and found that those with balanced, large-scale brain networks are able to moderate the cognitive consequences of threat [52]. Numerous studies have found that lower levels of education and/or cognitive abilities are risk factors that increase vulnerability to PTSD symptoms [53]. The ASD group in this study also showed lower levels of education and IQ than the HC group; however, the IQ score could be affected by an individual’s clinical state or psychiatric symptoms. Moreover, several deficits in executive functioning were still significant after adjusting for education and IQ level. Thus, it is difficult to explain the results in this study only using the predisposition of lower intelligence level. Our results describe the natural features of survivors with ASD rather than the conclusive causal factors.

We did not find significant correlations between survivors’ executive functioning and the severity of ASD measured using PSS-SR; however, psychological state regarding acute stress and comorbid symptoms including depression may also affect executive functioning [29]. Interestingly, for the survivors with ASD in this study, the level of state anger had a relationship with low reaction time before complex problem-solving. Findings regarding the relationship between anger and executive functioning in the ASD group are distinct from those from previous studies on later stages of trauma and PTSD, which have shown that depressive symptoms seem to mediate the relationship between PTSD and executive function [54]. With this phenomenon, it is plausible that the rape event upsets survivors and leads to attentional problems and impulsivity as a consequence of an unstable emotional state [55]. Loss of a sense of control and self-regulation also may affect survivors’ executive functioning [56]. Although not significant, a negative correlation was found between anxiety and planning ability. Taken together, a “flight,” survival response to stress may have influenced the ASD group’s short reaction time and may be associated with anxiety and state anger; however, that survival strategy seemed to be ineffective in the present study shown by the low accuracy levels associated with planning and other tasks. On the other hand, a positive correlation was found between reaction time in the inhibition task and depression in the ASD group, even though the ASD group showed shorter reaction time than the HC group. Future studies with larger samples are needed to more thoroughly understand the interplay and complex interrelationship among cognitive functioning and psychological variables.

Executive function not only controls and processes the trauma-related stimuli and responses through a top-down process but also interacts with the limbic system and other areas, affecting overall cognition including memory function in a long-term way [57]. Impairment of executive function might influence the development of psychiatric disorder or comorbidity including obsessive-compulsive and substance use disorders. Trauma from sexual assaults might also distort survivors’ cognition regarding self and interpersonal perspectives. It has been suggested that poorer executive function predicts worse therapeutic response as high-order cognitive functioning may play an important role in reconstructing and reintegrating the event, self, and others [58]. Even if survivors do not develop a psychiatric disorder, decreased executive functioning might lead to obstacles in daily recovery such as occupational functioning of survivors. Though it is not possible to determine whether the deterioration in executive function of a rape survivor with ASD will affect the progression of or transition to PTSD, the results of this study highlight the necessity of early intervention for sexual assault survivors. For example, neurofeedback or computerized training to improve executive functioning could be helpful for sexual assault survivors. Moreover, emotional state, especially anger, was found to be related to executive function in this study. This could indicate that various therapeutic methods promoting emotional stability, including medication, cognitive-behavioral therapy, and eye movement desensitization and reprocessing, should be combined with cognitive function-focused intervention. Future research on executive functioning and therapeutic prognosis is needed.

Depending on the type and intensity of the trauma, the effect on survivors may be different when it comes to developing/maintaining PTSD and overall socio-psychological health. Particularly, exposure to sexual assault for women has been found to be the main causal factors of PTSD [59]. Sexual assault especially is known to be more closely associated with decreased psychological well-being and satisfaction than other types of sexual harassment [60]. The present study contributes to the field of psychiatry and psychology because it focuses on an underrepresented group—rape survivors who recently experienced trauma—and, through its examination of clinical symptoms, emotional state, and cognitive function, it suggests possible therapeutic methods that could be used as treatment.

This study has several limitations that are mentioned below. We recruited sexual assault survivors who visited a medical center. For this method for recruitment, controls exposed to sexual assault without psychiatric diseases or survivors reluctant to disclose were not included in this study. Regarding the sample size, it was insufficient to validate and generalize the results. Additionally, several adolescents (*n* = 3) were included in this study, but due to their small number, more analysis concerning age was not conducted. We recruited age-matched HCs for this study; as different types of trauma, such as other violence and motor vehicle accidents, were not included, it is difficult to determine if the findings resulted from sexual assault or are a general effect of other traumatic events. Furthermore, this study is cross-sectional in design; therefore, the causal relationship of impaired executive functioning and predisposition based on cognitive functioning in ASD still remains unclear. More research on not only possible causal relationships but also fluctuations in outcome measures over time is needed. To clarify the issues mentioned above, a large-scale longitudinal study would be helpful.

## 5. Conclusions

This study found that survivors with ASD display global impairments of executive functioning after being exposed to recent sexual assault compared to controls never exposed to sexual assault. We have shown that these results might be from trauma-related effects in survivors with ASD, rather than being associated with low IQ and education levels. In addition, state anger level was found to influence survivors’ deficits in executive functioning. The findings stress the importance of assessment and intervention for sexual assault survivors in the early stages of trauma.

## Figures and Tables

**Table 1:** Descriptive statistics for demographic and clinical variables.

	ASD (*n* = 27)		HC (*n* = 25)		Wald *X*^2^	
	Mean	SD	Mean	SD	*F*	*p*
Age (years)	27.76	10.78	28.03	6.57	0.01	0.914
Education (years)	13.83	2.78	15.72	2.49	6.14	0.0132 *
IQ	103.71	16.19	113.24	8.37	6.58 ^1^	0.0138 *
Vocabulary	11.71	2.90	12.68	2.17	1.66 ^1^	0.2040
BDI-II	27.64	12.45	6.64	4.4	63.28	<0.0001 ***
BAI	28.36	15.2	2.84	3.05	67.76	<0.0001 ***
STAXI-S	20.32	8.44	10.72	1.14	54.72	<0.0001 ***
STAXI-T	18.96	5.78	17.88	5.04	0.50	0.4801
PSS-SR-total	37.2	9.27	-	-	-	-
PSS-SR-reexperience	10.36	2.91	-	-	-	-
PSS-SR-avoidance	4.92	1.38	-	-	-	-
PSS-SR-numbing	10.16	4.26	-	-	-	-
PSS-SR-hyperarousal	11.76	2.74	-	-	-	-
PSS-SR-dysphoria	16.88	5.25	-	-	-	-

* *p* < 0.05; *** *p* < 0.001; ^1^ *F*-test; ASD = acute stress disorder; HC = healthy control; BDI-II = Beck Depression Inventory-II; BAI = Beck Anxiety Inventory; STAXI-S = State Trait Anger Expression Inventory-State; STAXI-T = State Trait Anger Expression Inventory-Trait; PSS-SR = PTSD Symptom Scale-Self Report; IQ = intellectual quotient.

**Table 2:** Group differences in executive functioning.

	ASD (*n* = 23)		NC (*n* = 25)					
	Mean	SD	Mean	SD	Wald *X*^2^	*p*	*p* ^a^	*p* ^b^
IED-TE	20.52	12.89	15.96	10.49	13.72	0.0002 ***	0.0182 *	0.0228 *
IED-TT	83.70	20.21	77.92	17.54	1.13	0.2881	0.5623	0.6094
SOC-ITT (msec)	5929.26	4245.15 5	12,623.36	13,989.06	7.42	0.0064 *	0.0199 *	0.0508
SOC-PS	7.78	2.13	9.16	1.68	5.95	0.0148 *	0.4733	0.2262
SSP-SL	6.96	1.94	7.76	1.27	2.79	0.0948	0.3137	0.6396
SSP-TE	13.22	8.71	10.8	7.49	5.85	0.0068 *	0.0013 **	0.0066 ***
SST-PSS	0.52	0.14	0.62	0.15	5.34	0.0209 *	0.1636	0.0520
SST-RT (msec)	517.06	207.86	698.77	234.25	7.76	0.0053 **	0.0191 *	0.0066 **

* *p* < 0.05; ** *p* < 0.01; *** *p* < 0.001; *p*^a^ = education adjusted; *p*^b^ = IQ adjusted; ASD = acute stress disorder; HC = healthy control; IED = Intra-Extra Dimensional Set Shift; IED-TE = IED total error; IED-TT = IED total trials; SOC = Stockings of Cambridge; SOC-ITT = SOC mean initial thinking time on five moves (msec); SOC-PS = SOC problems solved; SSP = Spatial Span; SSP-SL = SSP span length; SSP-TE = SSP total errors; SSP = Stop-Signal Test: SST-PSS = SST proportion of successful stop in last half trials; SST-RT = SST mean correct and incorrect reaction time on go trials (msec).

## Supplementary Materials

The following are available online at http://www.mdpi.com/2077-0383/7/10/362/s1, Table S1: Spearman’s correlations in the ASD group.
