# Supplementary material for: Impaired Executive Functioning of Sexual Assault Survivors with Acute Stress Disorder
Source: J Clin Med. 2018 Oct 16;7(10):362. doi: 10.3390/jcm7100362 (PMC6211069; doi:10.3390/jcm7100362)
Supplement: Supplementary file 1 [file jcm-07-00362-s001.pdf]

Table S1. Spearman's correlations in the ASD group.

Variable	Variable	*Rho*	*p*	*q*	Rank
PSS-SR-numbing	PSS-SR-dysphoria	0.94	0.000000	0.000292	1
IED-TE	IED-TT	0.93	0.000000	0.000585	2
BAI	PSS-SR-hyper-arousal	0.82	0.000000	0.000877	3
PSS-SR-total	PSS-SR-dysphoria	0.80	0.000002	0.001170	4
PSS-SR-total	PSS-SR-re-experience	0.77	0.000005	0.001462	5
PSS-SR-total	PSS-SR-hyper-arousal	0.76	0.000010	0.001754	6
PSS-SR-total	PSS-SR-numbing	0.74	0.000023	0.002047	7
BDI-II	PSS-SR-total	0.72	0.000055	0.002339	8
BDI-II	BAI	0.71	0.000060	0.002632	9
BDI-II	PSS-SR-dysphoria	0.70	0.000104	0.002924	10
SOC-ITT	SOC-PS	0.71	0.000146	0.003216	11
BDI-II	PSS-SR-hyper-arousal	0.68	0.000186	0.003509	12
BAI	PSS-SR-total	0.67	0.000236	0.003801	13
BDI-II	STAXI-S	0.67	0.000259	0.004094	14
PSS-SR-hyper-arousal	PSS-SR-dysphoria	0.67	0.000283	0.004386	15
BDI-II	PSS-SR-numbing	0.64	0.000546	0.004678	16
PSS-SR-total	PSS-SR-avoidance	0.60	0.001472	0.004971	17
BAI	PSS-SR-dysphoria	0.56	0.003381	0.005263	18
BAI	PSS-SR-re-experience	0.56	0.003798	0.005556	19
BAI	STAXI-S	0.56	0.003846	0.005848	20
STAXI-S	PSS-SR-numbing	0.55	0.004422	0.006140	21
SOC-ITT	STAXI-S	−0.57	0.004528	0.006433	22
STAXI-S	PSS-SR-total	0.54	0.005222	0.006725	23
STAXI-S	PSS-SR-dysphoria	0.54	0.005317	0.007018	24
IED-TE	SOC-ITT	−0.55	0.006436	0.007310	25
PSS-SR-re-experience	PSS-SR-hyper-arousal	0.52	0.007793	0.007602	26
BDI-II	PSS-SR-re-experience	0.52	0.007954	0.007895	27
IED-TE	SOC-PS	−0.52	0.010570	0.008187	28
IED-TE	SSP-SL	−0.51	0.012736	0.008480	29
IED-TT	SOC-ITT	−0.51	0.013062	0.008772	30
PSS-SR-avoidance	PSS-SR-numbing	0.49	0.013260	0.009064	31
BDI-II	SST-RT	0.50	0.016534	0.009357	32
IQ	SOC-PS	0.54	0.017793	0.009649	33
STAXI-S	SOC-PS	−0.49	0.017964	0.009942	34
SST-RT	SSP-TE	0.49	0.019517	0.010234	35
STAXI-S	PSS-SR-re-experience	0.46	0.020186	0.010526	36
STAXI-S	PSS-SR-hyper-arousal	0.46	0.021464	0.010819	37
PSS-SR-avoidance	PSS-SR-dysphoria	0.45	0.023240	0.011111	38
PSS-SR-numbing	PSS-SR-hyper-arousal	0.45	0.023565	0.011404	39
IED-TT	SOC-PS	−0.47	0.023792	0.011696	40
STAXI-S	STAXI.T	0.44	0.026615	0.011988	41
PSS-SR-re-experience	PSS-SR-avoidance	0.44	0.028401	0.012281	42
BDI-II	STAXI-T	0.43	0.031891	0.012573	43
PSS-SR-avoidance	PSS-SR-hyper-arousal	0.42	0.037972	0.012865	44
BAI	PSS-SR-numbing	0.41	0.041704	0.013158	45
BDI-II	PSS-SR-avoidance	0.41	0.044053	0.013450	46
PSS-SR-re-experience	PSS-SR-dysphoria	0.40	0.044862	0.013743	47

Only correlations with *p*-values less than 0.05 were presented. False discovery rate based on the Benjamini and Hochberg method was used for multiple testing corrections. The correlation was considered significant if the *p*-value was less than the *q*-value. ASD = acute stress disorder; BDI-II = Beck Depression Inventory–II; BAI = Beck Anxiety Inventory; STAXI-S = State-Trait Anger Expression Inventory-State; STAXI-T = State-Trait Anger Expression Inventory-Trait; PSS-SR = PTSD Symptom Scale-Self Reported; IED = Intra-Extra Dimensional Set Shift; IED-TE = IED-total error; IED-TT = IED

total trials; SOC = Stockings of Cambridge; SOC-ITT = SOC mean initial thinking time on 5 moves (msec); SOC-PS = SOC problems solved; SSP = Spatial Span; SSP-SL = SSP span length; SSP-TE = SSP total errors; SSP = Stop-Signal Test; SST-PSS = SST proportion of successful stop in last half trials; SST-RT = SST mean correct and incorrect reaction time on go trials (msec).
